# Supplementary material for: Telomeres reforged with non-telomeric sequences in mouse embryonic stem cells
Source: Nat Commun. 2021 Feb 17;12:1097. doi: 10.1038/s41467-021-21341-x (PMC7889907; doi:10.1038/s41467-021-21341-x)
Supplement: Supplementary file 1 — Supplementary information [file 41467_2021_21341_MOESM1_ESM.pdf]

Supplementary Information

Kim et al.

Telomeres reforged with non-telomeric sequences in mouse embryonic stem cells

Supplementary Figures

Supplementary Fig. 1: ALT mESC does not show ALT cancer-specific features.

Supplementary Fig. 2: mTALT of chromosome 11 is used for ALT telomere maintenance.

Supplementary Fig. 3: A complete sequence of the mTALT of chromosome 11.

Supplementary Fig. 4: Genomic features and chromatin landscape of ALT mESCs.

Supplementary Fig. 5: TRF2 is necessary for telomere maintenance of ALT mESCs.

Supplementary Fig. 6: Specific Gene Ontology terms are enriched in ALT mESCs.

Supplementary Fig. 7: HMGN1 is required for telomere maintenance of alternative lengthening of telomere (ALT) mESCs.

Supplementary Fig. 8: Telomere maintenance is distinctively affected by stemness genes, HMG family genes and RNaseH1.

Supplementary Data

Supplementary Data. 1: List of differentially expressed proteins.

Supplementary Data. 2: List of differentially expressed genes.

Supplementary Data. 3: List of used primers.

Supplementary Fig. 1

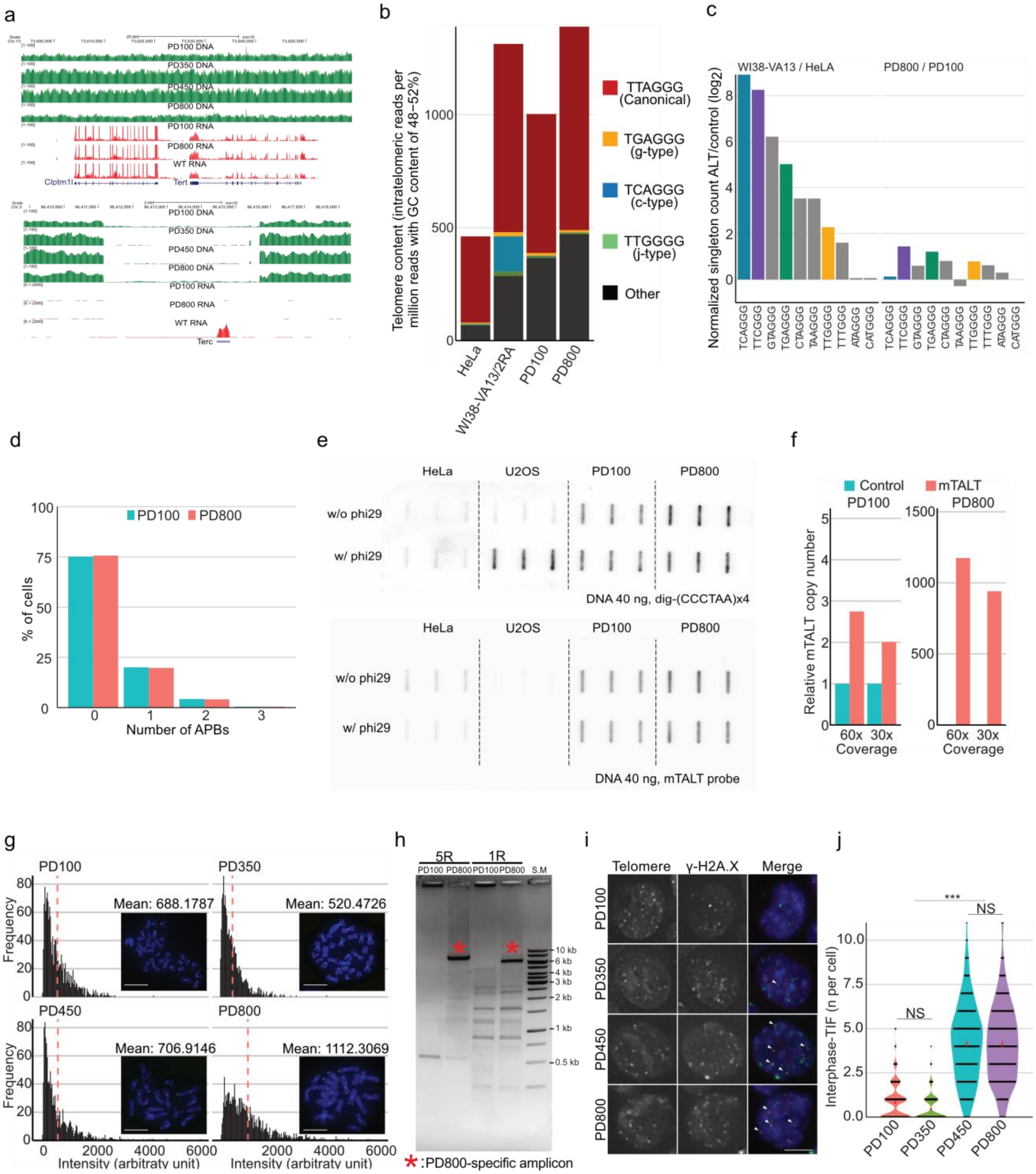

* :PD800-specific amplicon

Supplementary Fig. 1

ALT mESC does not show ALT cancer-specific features. **a**, Genomic snapshot and RNA expression of the Terc (left) and Tert (right) gene. **b**, Quantification of canonical and variant telomeric repeats. Using the 'Telomere hunter' software, the compositions of variant repeats were estimated from WGS data. **c**, Quantification of singleton telomeric repeats of PD800 cells normalised to PD100 cells. **d**, Bar graph showing the percentage of cells with APBs. **e**, C-circle assay. Dot-blot result with telomeric C-strand- or G-strand-specific probes. C-circle assay was performed 3 times with similar results. **f**, Calculated copy number of mTALT from WGS according to sequencing coverage. Coordinates of the control region are chr13:109998778-110006148. **g**, Telomere length quantified with quantitative FISH. Scale bars, 5μm. **h**, PCR assay showing that post-ALT cell has translocated mTALTs in chromosomes other than chromosome 13. 1R and 5R indicate the right subtelomere of chromosome 1 and 5, respectively. **i**, Representative images of TIF analysis. Scale bar, 5μm. **j**, Violin plot showing the TIFs at each time point. Each dot represents TIFs in a cell. The dots and bars represent means and confidence intervals for the means, respectively, from ≥98 cells per condition over three independent experiments. *P* value from a two-tailed unpaired t-test: *** $P < 0.0001$, NS, non-significant. Source data are provided as a Source Data file.

Supplementary Fig. 2

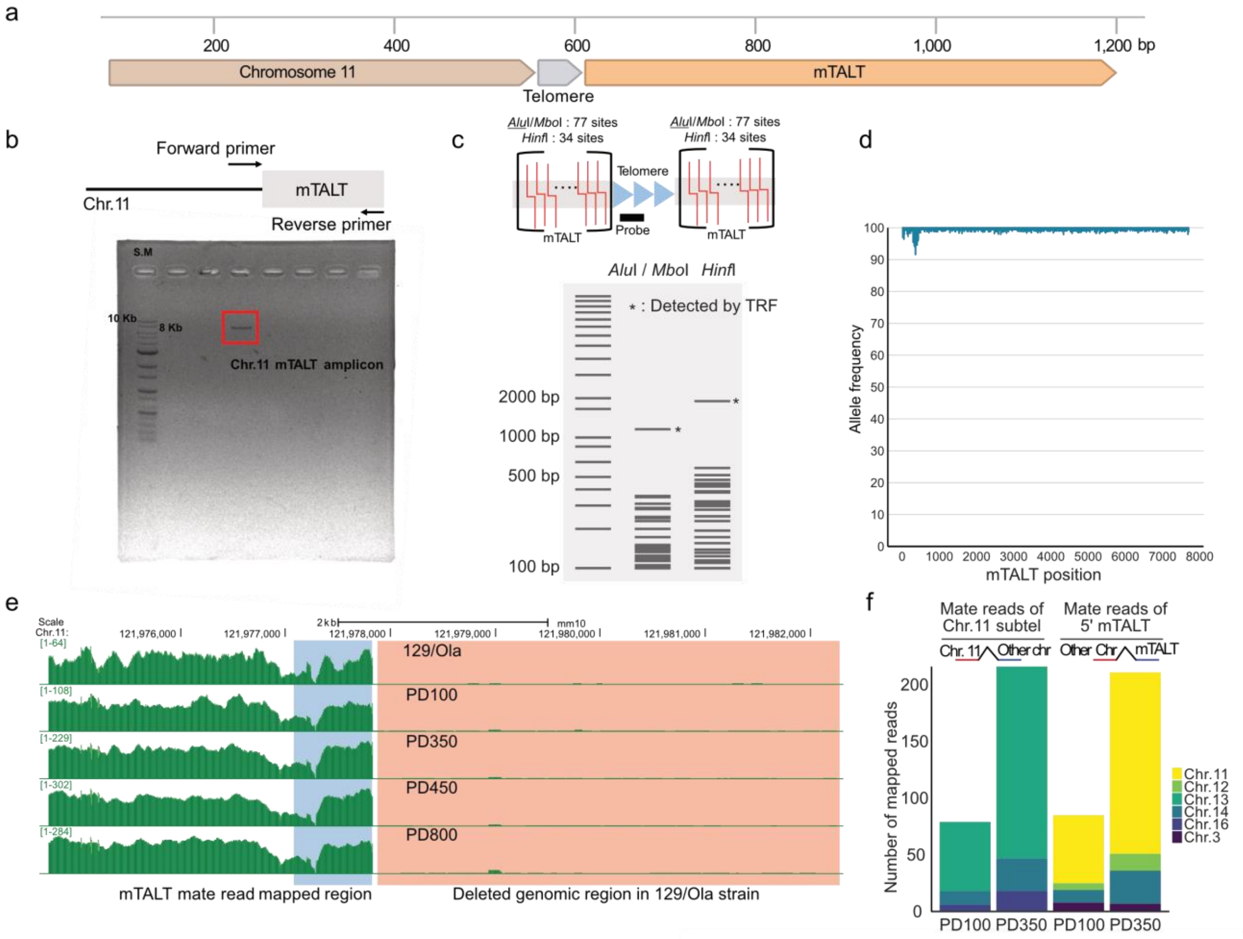

g

Location	PD100	PD350	PD450	PD800
Passed Filters	206	185	128	129
SNP	185	168	120	121
Insertions	7	5	2	2
Deletions	14	12	6	6
SNP Transitions / Transversions	1.53 (130/85)	1.45 (122/84)	1.50 (144/96)	1.48 (144/97)
Total Het / Hom ratio	5.87 (176/30)	3.63 (145/40)	0.00 (0/128)	0.01 (1/128)
SNP Het / Hom ratio	5.38 (156/29)	3.42 (130/38)	0.00 (0/120)	0.01 (1/120)
Insertion Het / Hom ratio	Inf (7/0)	4.00 (4/1)	0.00 (0/2)	0.00 (0/2)
Deletion Het / Hom ratio	13.00 (13/1)	11.00 (11/1)	0.00 (0/6)	0.00 (0/6)
Insertion / Deletion ratio	0.50 (7/14)	0.42 (5/12)	0.33 (2/6)	0.33 (2/6)

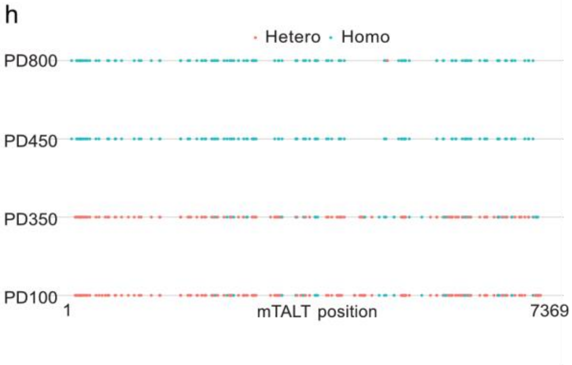

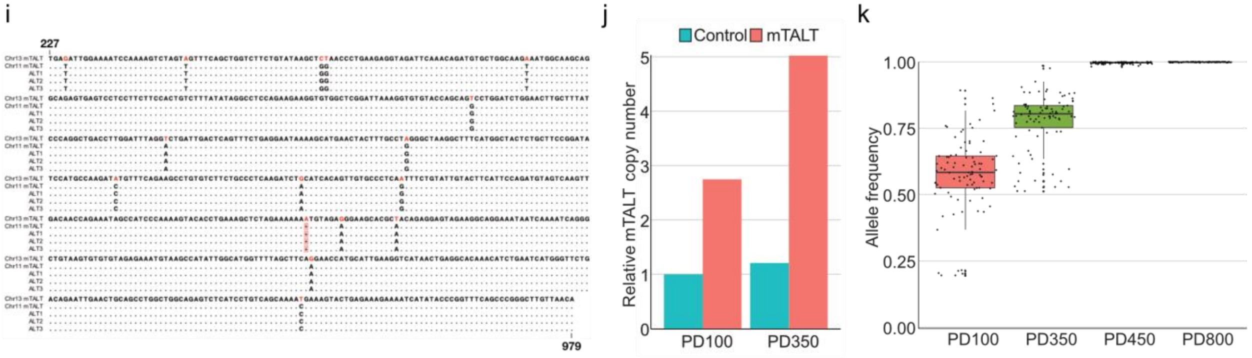

Supplementary Fig. 2

mTALT of chromosome 11 is used for ALT telomere maintenance. a, Schematics of the putative genomic structure of the right subtelomere of chromosome 11 in ALT mESCs. **b**, Schematics of PCR to amplify the mTALT of chromosome 11 and a representative result. **c**, in silico digestion result of the mTALT of chromosome 11 with specified restriction enzymes. **d**, Allele frequency of the mTALT of chromosome 11 for each nucleotide position. **e**, Snapshot of WGS reads of the right subtelomere of chromosome 11 in ALT mESCs. ~5kb of the terminal region of chromosome 11 was deleted. **f**, Mate reads analysis: (left) Locations of the mate reads of the reads mapped to the indicated region in b which is the terminal end of chromosome 11. (right) Locations of the mate reads of the reads mapped to the mTALT region. **g**, Summary of SNP analysis of the mTALT region. The reference genome used is mm10. **h**, Locations of either type of SNPs throughout the mTALT sequence. **i**, Comparison of SNPs of the mTALT region among independent ALT mESCs. ALT1, ALT2 and ALT3 are independently obtained ALT mESCs from reproduction experiments. **j**, Calculated copy number of mTALT from WGS. **k**, Allele frequency analysis of the mTALT region to differentiate the SNP types of PD100 and PD350. Data are presented as box-whisker plot overlaid with dot plot (individual data points). In box-whisker plot (89 SNPs were tested in each sample), centre line, box, and whiskers denote median, the interquartile range (IQR), the rest of the data distribution, respectively. Source data are provided as a Source Data file.

Supplementary Fig. 3

GCTGGGGTTCCCAGGTCCACAGCACCAGGGGTGCTGGCATGAGTGATGTCTCAAGGCCTCAAAATTATCCATGGCCCACGCCCATGCCTG
TTCTCGGGTCATGTCTGGTGGCATGACCAGGTCTGGTTGGTACATCCTTCCAGGCAGCTCTTCCAAGGGTGATGCAACTGAAGACATGGCT
TCTGCTTCCCAAGCAAGTAGCAGAGATTAGCAGCTGAGGGGCAACTGATATTGGAAAATCCAAAAGTCTAGTTGTTTCAGCTGGTCTTCTGTA
TAAGCTGGAACCCTGAAGAGGTAGATTCAAACAGATGTGCTGGCAAGTAATGGCAAGCAGGCAGAGTGAGTCCTCCTTCTTCCACTGTCTTT
ATATAGGCCTCCAGAAGAAGGTGTGGCTCGGATTAAAGGTGTGTACCAGCAGGCCTGGATCTGGAACTTGCTTTATCCCAGGCTGACCTTG
GATTTAGGACTGATTGACTCAGTTTCTGAGGAATAAAAGCATGAACTACTTTGCCTAGGGCTAAGGCTTTCATGGCTACTCTGCTTCCGGATAT
CCATGCCAAGATATGTTTCAGAAGCCTGTGTCTTCTGCCCTCAAGATCTACATCACAGTTGTGCCCTCAGTTTCTGTATTGTACTTCATTCCAG
ATGTAGTCAAGTTGACAACCAGAAATAGCCATCCCAAAAGTACACCTGAAAGCTCTAGAAAAAAATGTAGAAGGAAGCACGCAACAGAGGAG
TAGAAGGCAGGAAATAATCAAAATCAGGGCTGTAAGTGTGTGTAGAGAAATGTAAGCCATATTGGCATGGTTTTAGCTTCAAGAACCATGCATT
GAAGGTCATAACTGAGGCACAAACATCTGAATCATGGGTTCTGACAGAATTGAACTGCAGCCTGGCTGGCAGAGTCTCATCCTGTCAGCAAA
ACGAAAGTACTGAGAAAGAAAATCATATACCCGGTTTCAGCCCGGGCTTGTTAACATCAGAGCAGAATTTTGCTTGGCAGCAAGTAGAAAGC
ACTCTCTAACAGGGATGGTCAGCAAGGACTCTGGACATCTGAAAATGGCCCAAGGCACACTGCTGGCTCCTGAGCGACCTCTACTGTGAAA
AAGTCACAGGCTTGCAGCTTGGAGCTAAGAACCCTTTATCTTTCTGTCTAGACTCATTTATTGGCCAGGCTCACTTTCTGGGATCCACCAGTG
TGGACAAGCAAGCAGGCTTCTAACTATGGACTTGTCCAGGATCACAAAGCTGGCTCCAACCCCTGGGTGTGCGAGTGAGAAGCCTTCAGTT
AGTGTCTTCTGGCATGTTACACGCTGAACAAGTGCACACCAGGGCCATAGCCATGGAGGGACTGCAGAAAGGAAGGCATGCAGGAGAAAG
ACAGCTCAGAGAAATACAGCTTAAGCGTCCTGGGCAATGCTATCCTGGAAAAACCTCTTCACAACAAAAGGAACGCAGCTTCAACAGAGATT
CCAGCAGACAGCAATTTTGTAGACCTCCATAGGTGGGCAGCAGTGGGATGGAAAGCGAACCAAGGACAGCTTCGCCATACATTCAGTAATG
AAAAGAAATTGGACGAATTTTCAGCGTTTAGAAACGAGGAAGAGCGGAGAGGACACAAGACGATCAGAAACCGCAGGGTCTGCATCCCGTC
TCCTGCAGACTCACACCTGGGCGCCAGGCTTTCTGTCAGCATTTAGGAGCACACCATAAGTTCAAAAAAATGGAATCCCACCACAAACAGAA
TCGGATCTGGTTGACAAAACGAAAGAACAGAGAAACAAAATCACGTACGCGGTTTGAAATGACCATCGGATTTCAGTCAGAGAGAGCATGCT
GACAGCCAAGAACTTGACTCTATGAGAAAGTTGCAGTATTTGCTCCCTGAGGATCCAAAGGCGGGAAGTTTACCATTAATTGGCATTCCTCTC
TGGGGGAAGAGCAGACCCACAGCGACCCCTGCTGTGAAGTCGTGGCCAGACTGGAGCTCAGAGATAGAGCTTTGAGGCTTCTGGCTCCTT
CATGGGTCCCCACTCCAGCTGTGGCCCCTACACGTTTGGAAAAGGGGGGGACCAACTATGGATTTTCCCAGGATCGCACGACTCGCCTAA
AACTCGGGGTGTGCGAGAGAGTTAACCGGGTGGTCCAGTGCTTTCCCTGTTGGACAAGTGTACTCTTGGGCCATGGCCACGAAGCAACCA
GGGAAACTACAGTGAGATGGACAGACAACCTGAGCTTGAAAGGCCTTAGGTAAACAGAAAGGAAAACAGCTGCGATGGAGAGTTGTGTTTG
CACTCCGTTTGTCTTCTTCAGGGGAAGAGGGCGGTGCTGCAAGTCTTGGCACACTTGGTGGGCTAAGAAGATGGTATGTAATCTAAGTTTTG
AAACGCGGGGAGGTCGAGAGGAGCAGAAAACAAACCAAATCCGCGGAACCTGAATAGCATCTTCTACATCCCCGCACACATGCGCCAGGC
ATTTTGGTCAGGATTTAAGTGCACACCTTCCCGACCTGAAGTCAGCACAGAATCCCACCTCCCACTGCTTGACAGAGTCAAATCCTGTCAGC
ACAGCGAATGTACAGAGAAACAAAATCATGAACGACCTTTGAAATCACCTTCGGATTTCAGCTCCAGACTTCTGACAACCCTGTGGAACAAG
GGATACAAGTTATAGCTACTAGAGAAGGGTGTCCAGAGTGTGAGCACCAAGGTTGCCACCAGCTGGAGTGCCAGCACCTCAGCGACCCCT
GTTGTGAAGAAGTCTCTGGGCTGCCCCTCTGAAGAAGAACTGTGGATCTGCCAATGGAGACTATTTGGCTTGTTGCCAGGACTCCTTCCTT
GGCTCTGGCTCCTGCATTTCCCTGGAACCACCAAAACCATCCTGCATGACAGCGGTTCATTGGAGGGGACCAAGCTAACTCAAATGAAATAA
AGAACCTACTATTAGTTATACTATATTGGCGCATGTCTGTTATTTTTGGAGATAACTAACATTTCCCTGGCACTACAATTCTCTTCTTACCCCTCG
CCTCCTTGGTGTGTTGTTTCTGTTTTATATACTCTATAAGCAATTCTGCCTTTTTCACTTCTTTGTCTATGTTGTGGATTTGTAAACACCAGATTT
CAGGGTCCTTTGCTTGCTTCCCCCACCTCTTTCTGGATTACAAATTCCACCCACAGTGTGACTACAGAAAGTTTATGTGAGCCATCTCAGAAT
ACACACTGCTATTGTCCTGTGTGCCTGGGCCTAGAGCAGTAGAGCTGGCTCTTTGGTCCTGTGTGTGAAGAAGCTGTCCTGGGATTCTGCA
CCAGTGTCTCCTGGCCTCGTCCAGTACTAACATGAGCAGATTTTGAGCCATGGCTTTCTGCCCTTGACATCTGTACTGGCTAGTTTTGTGTCA
ACTTGAGTTATCTCAAAGAAAGGAGCTTCAGTTGAGGAAATGCCTCCATGAGATCCAACTGTAAGGCATTTTCTCAATTAGTGATCAAGTGGG
AAAGGCCCCTTGTGGGTGGGACCATCTCTGGGCTGGTAGTCTTGGGTTCTATGAGAGAGCAGGCTGTGCAAGCCAGAGCAAGCAAGCCAG
TAAAGAACATCCCTCCATGGCTTCTGCATCAGCTCCTGCTTTCTGACCTGCTTGAGTTCCAGTCCTGACTTCCTTTGATGATGAACAGCAGTA
TGGAAGTGTAAGCCAAATAAACCCTTTCCTCCCCAACTTGCTTCTTGGTCATGATGTTTGTGCAGGAATAGAAACCCTAACTAAGACAACATT
CCTGACTTAGAAGGTCTCTCATTTAATTCTCTTTTCTCATGTTATGAATCTGCGGGGCTATCTCAGGAAGTACAAGATCTCAAATACATAGGGA
GAGTAGTGAAAACAGGGGAAAATCCTCCATATTTGCTTATTATCTAGTCCAGGTCCAGGGGAACTGAACTGAAGAGGCTTCTACTTGTTTAAAT
ATACACTTTTCAAAATATTACAATGCCCAGAATCCAATCAAGGCTTATATGAGAAAGTGCTGAACTATGAGTTTCAAAATAATCCCCATTAAACTT
TGATACCAAATTATTTTGACAGTCCCTTAAAAACAGCTTTCCAGTCTTTCTGCCTGCTTCCCTGCAAATATGAATCCAGTTCAGGGCAGCCTGG
AGCCCAGCTACTCCAGCAGATCCCTGTCTGCCTTCTGTCCAGACACAAAGACTAGAGGATGGAAATGTTACCTGCATTCATCTCAAACACAT
GCATCGAAAACAGGGCTGGGGGAGTGGGCGGGTGGAGAGGAGATTCCCTGTCCTGAAGTTTCATCTCTACACTTAGCTGTTTCCCCTAACC
ACTTCTCTGGTGGGAATAGAGGATTAGCATCTGCTCAGGACTCCAGCTGACCTAAGGACTATTAACAGGATTTCCAAGCAGGACCTGGTGAC
TCATGCCTTTAATCTCAACACAAAGGAGACAAGGCAGGGAGACTTGACTTGGTATCCAGCTTGGTCTACAAAGTGATTTTCATAAGACCCAGT
GTTACATAGAGAAACACTCTCTGTGTTGGCTATTGTTTGTTATCAACTTGGTTATCTCTGCAATTAAGCAAAATCCAAAATGGGAAGATACATCT
GTAAGTAACTTTTGCTTAATTTATAGAAAGTAATCCATACCTGTGAGGTAGGAAGACTTGCTTTTAATGCAGATCTTTTTGAGCTTGAAAGACAA
GGCCTCCAATTAGGATCTGAAGCCCGAAAGACACACTTTTTTGTTAATGTGATTGTTGTGATAAGCTAGGGTTTGTCATTAATACCCCTGGCAG
TCCTGTAACTCACTTTGTAGAGCAGACTACTCACAGAGATCTACTTCCTTGATTCAATTCCATAGTCCTTGGATTAAAGGCGTGAGCCACCAC
ACTTAGCATAAGACACTGAACTTTAATCCAGAGGTTGAGGCTGGAACCACACACCTTTAATTCTGGCCACACCTTCAGCTGGAGGCCTATATA
AGAACAGGGAGGAAGAGAGTTTTCACTTGGCCTGCTTGCTCTCTCATAGCTAACAAGTCTATTACTTCGCTGGCACTAGAGCCCCCTTGCAT
GTGATTCCATCTTCTATTGAAGACCAGCTGAAACATCCATCCTCAGGAACTGAGAACTTCTGGAATCTTAGACTTTACTTCCTCTCCAGCTGTT
GTGGAATAAGTTCAACTGCAGCCTGTAAGTCATTCTAATAAATCCCCTTCCAGGAGAGAGAGAGTGAGGGATCCTTATATTTAGTATTTTACTC
TCTACACCAGGCAGCCCAAGTAGATTTGATGGTCCATTAGCACTTTCTCCATTGCCTGTTTAGTCAGCTGCCTATAATGTTTAACTGTAGACAA
TTGCATCCAGAGTGATAACATCTTACTTGGAGAAGCTTCACTCCTTTTTGTTTACTGGTTTTGATTTTTGAGCCTAGGTTTTCCTGTGTAGCACT
ACCTGATCTGAAACTAGGTCTGACAACCAGGCTGGCCTTGAGATCAGAGACCCATCTGCCTCTGCTTCTGTGTAGGATCTGCTCTTTCCCTT
AGGTTCTGGGATGAAAGGATTTTGCCACCAGATTGGGCAGAATCTTGTCTCCTTAGGCAATGCAAATAAGATTCTAGGTTTGTAGGTTTCAAA
CCCAGCTTCCCACAGTGTAATGGTTGGGAAGGGTCAAGAAATATAGAGCACAATTAACTTGATTCTTCTCTGAATCAGGAGGTGGAGGATTTA
CCTTCAGGTATTCATGGATAAAGCCAAGAAGATGATGCAGTCCATTCCCAGTTTTGTCAAGGATACATCAGATATTGAAGAACATGCACTGCC
CAGTGCACAGGTCTTTCCAGCCCTGAGTACAAGGTGTTCCAATTCTGAGACACTTTGTTTCAGCAAAGAGCAAAGCCACTGCTCTGAGGAT
GGCTGGATTGCCAATTGGGATCTATACTCCTTTTGTGTATTTGAGAGTGTGGACTACCTGAGATCCTACCACAGATTGAATTCTGCCATGAAG
AAGGGCACAGAGGTCTTCCAGAGTGAGAATCAAAGGGAGCCACAAGTGTCCCCAGGAGATGTGGAAAACTACAAAGACAAAGATACAGAG
GAGCCAGACCAACCCTCACTAAGCTTGCTCAGGGAGAAAGAGCTGGAACTTGTGACCTGTGATGGTGGAGACTGCCCTGACAAGGATCCT
GCATCTTACAGTGCCAGGCACCTAGGCTGCTGGACATGGCTTCAAAGAGCTTTTCGCCAGAAGTGAGAAAGCCACCCAGAACTGTTAGGAT
CCCAGATTCCTGCTAAGACTTGCAATTAGGGGATCTTCTGTCAGCTCCTGCTGGTACAGCAAAGGCACACAAAGGCAGTTGTGTCTTTTCAG
CCATCTGGTTTGTGTTTGTTTGTTTGTTTATTTGTTTGCAGCATTCTTAATAAAATTGTTAAAAAGCTGAAAATTTGTGTTCTTCTTAAAGTGGCC
CAAGGGAAAAGATAAAGGGTGTAGTGGACACAGATGAGGATATTCTATTTTTCCTACTGTTGGTTACAGGATAACTGGTGGCTTGTTTTAAAG
GAAGTCCTTAGGGGATGTTAGGCCTCCTTGTTCTCCTTTGTGCTGGGCCATAAGATAAACACACAGGGAGCAACTTTGAGCACTTTGGGAAA
CCTTCCTGCTGAATCACCATGGATGATGACTGATCTACATAGATGCTTCCCTTCCAAAAAGATCTAGGGGACACCTGCAACTTCCATTTTTACT
CTTTTACCTTATGCATAGACTAGGCTAGCTCTGAACTCTGATTCACTTTGCCTTGCTTCTCTATTATGGGGATTAGAGGCCTGTGTCAGCACAT
CTGCACAGGAGAATTTTTCTATTCTCAGCAATTTCCAGAATAAATTTCTACATCATATGTGAACTCACCTCCAGAAAAAGGACTGGCTAAGTTA
GAAGGTCAGGGCAGTGCTGGTCCTGACAGAGAAAACTCAGCCAACTTGAGAGGGTGTCTTTTGCTTCCAGTCTTGGAAGAGCTTAACATTT
TATCAGACATTAGTTCAGCTGTTGGTCTTCCAGAAGTCTAGCCTTTCAGTAGTAAATGGATCTGAGGTAGGCATGTTCACTCAGGCAGGTGTA
TCCCTGACAGTTTAGGGCAGCCTGATTAACACAGGGAATTCTAGGACAGACAGCATTATGAAGTGAGAGGCACAAATAAAAGAAAGAAGGGA
AGGAGGGTGGAAGGCAGAAAGGAAGGAAGTAATAGAGGGAGCGTGTGAAGGAGGAAGAGAGAAGGAGGGAGAGAGGGAGGAAAGAGGG
AGGACTGAGTGATGGACAGGTCTGAATGACAGGCCTCACTGCAGTTGTGGAGAATAAATGGAAGCAGCTA

Supplementary Fig. 3

A complete sequence of the mTALT of chromosome 11. The sequence was assembled based on the sequencing result of the mTALT PCR product.

Supplementary Fig. 4

a

Location	PD100	PD350	PD450	PD800
PassedFilters	3,884,052	3,851,713	3,867,249	3,856,131
SNP	2,340,028	2,307,847	2,320,113	2,311,947
Insertions	759,129	762,876	763,085	761,365
Deletions	775,353	777,624	778,368	776,703
SNPTransitions / Transversions	1.23 (2267987/1844406)	1.23 (2333402/1893558)	1.23 (2312678/1878344)	1.23 (2304093/1871330)
Total Het / Hom ratio	0.27 (830009/3045195)	0.15 (511813/3337263)	0.19 (626912/3235442)	0.20 (642951/3207956)
SNPHet / Hom ratio	0.32 (569324/1770704)	0.20 (390424/1917423)	0.24 (450953/1869160)	0.24 (450158/1861789)
Insertion Het / Hom ratio	0.20 (128821/630308)	0.08 (59122/703754)	0.13 (86205/676880)	0.14 (94425/666940)
Deletion Het / Hom ratio	0.20 (131170/644183)	0.09 (61538/716086)	0.13 (88966/689402)	0.14 (97476/679227)
Insertion / Deletion ratio	0.98 (759129/775353)	0.98 (762876/777624)	0.98 (763085/778368)	0.98 (761365/776703)

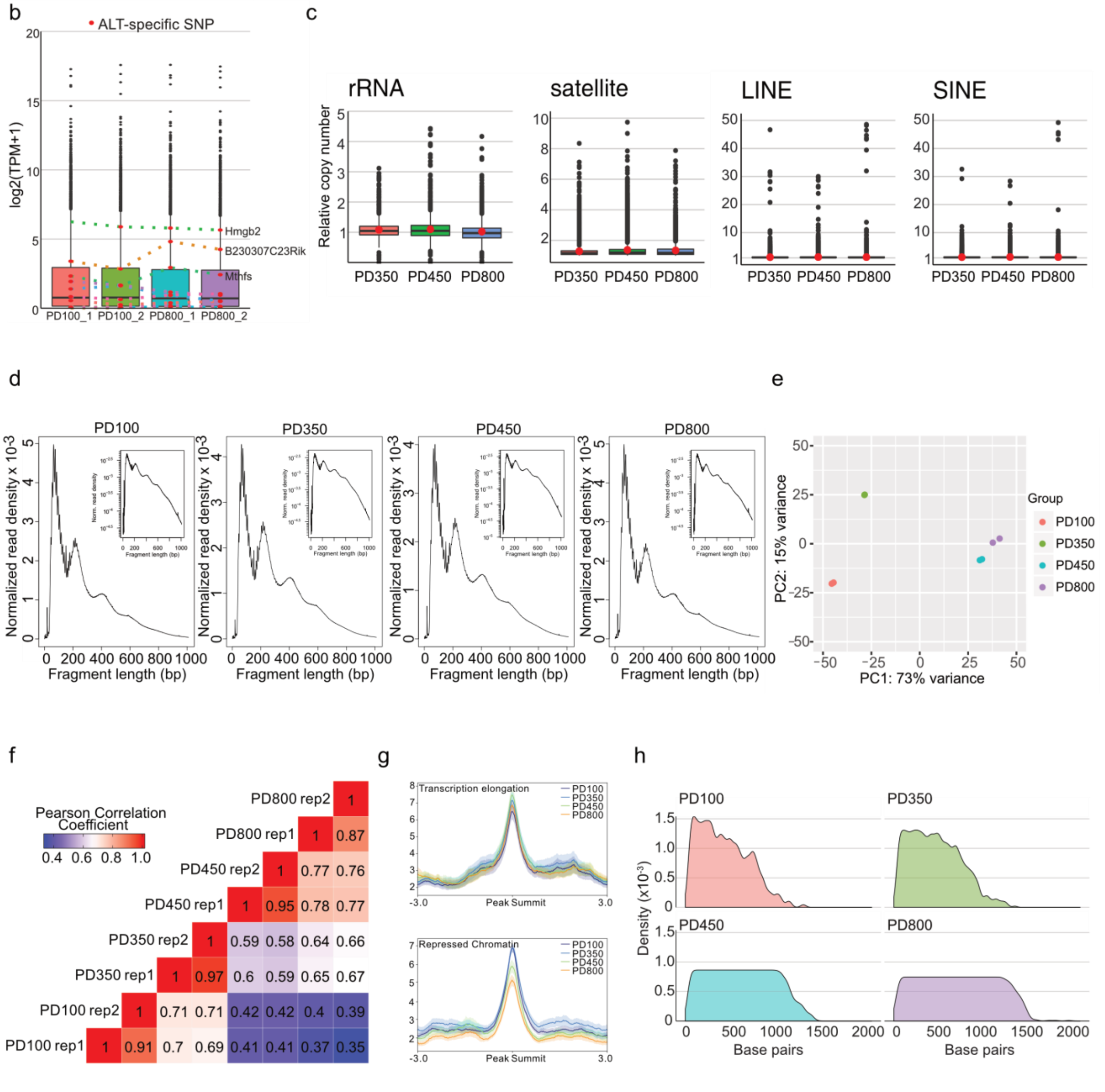

Supplementary Fig. 4

Genomic features and chromatin landscape of ALT mESCs. a, Summary of genome-wide SNP analysis. The reference genome used is mm10 of C57BL/6J. **b**, Box-whisker plot of differentially expressed genes. Red dots denote genes with ALT-specific SNPs. In box-whisker plot (n = 28182 PD100-1, n = 27483 PD100-2, n = 27218 PD800-1, n = 26844 PD800-2), centre line, box, and whiskers denote median, the interquartile range (IQR), the rest of the data distribution, respectively. Outliers greater than ±1.5 × IQR are denoted by dots. **c**, Quantification of various repetitive sequences at each time point compared to PD100. In box-whisker plot (n = 4686 rRNA, n = 114324 satellite, n = 2941137 LINE, n = 4616073 SINE) centre line, box, and whiskers denote median, the interquartile range (IQR), the rest of the data distribution, respectively. Outliers greater than ±1.5 × IQR are denoted by dots. **d**, Fragment size distributions of ATAC-seq data showing nucleosome periodicity. **e**, Principal component assay of ATAC-seq data at each time point. **f**, Heat map of differential ATAC-seq peaks. **g**, Normalised differential ATAC-seq peaks aligned to the peak summit. (Top) Differential peaks aligned to transcription elongation term-related regions are represented. (Bottom) Differential peaks aligned to repressed chromatin term-related regions are represented. The solid lines and the error bands represent the mean of each data and the standard errors of the mean, respectively. Source data are provided as a Source Data file. **h**, Fragment size distributions of ATAC-seq reads mapped to mTALT region. Source data are provided as a Source Data file.

Supplementary Fig. 5

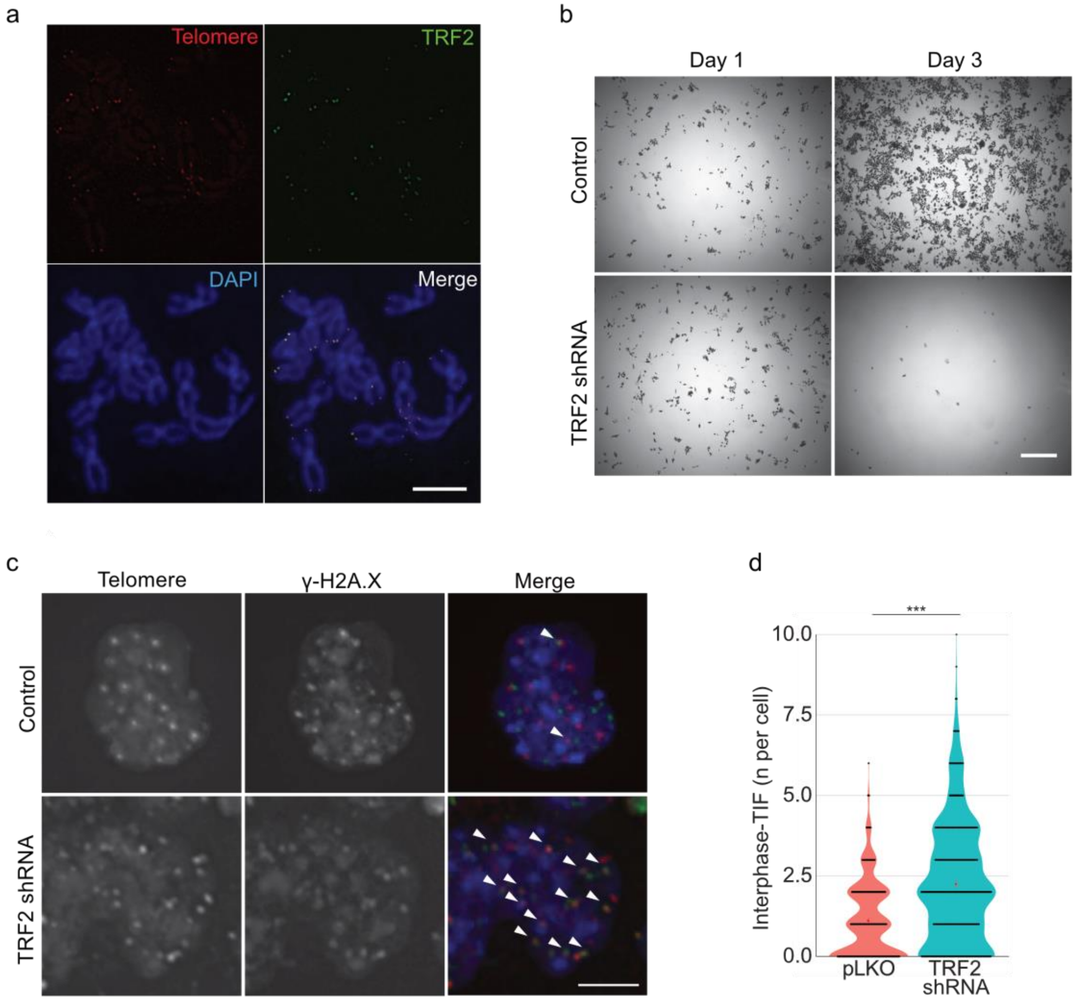

Supplementary Fig. 5

TRF2 is necessary for telomere maintenance of ALT mESCs. a, Co-localisation of TRF2 and telomeres of ALT mESCs. Telomeres are labelled with PNA (TTAGGG)4-Cy3 and TRF2 is stained with specific primary antibody and secondary α-mouse Alexa488 antibody. Scale bar, 2 μm. **b**, Growth rate assay of control and TRF2 knock-down cells. Representative images were taken from ≥20 images per condition over three independent experiments with similar results. Scale bar, 30 μm. **c**, Representative image of TIF analysis of the control and TRF2 knock-down cells. Scale bar, 5μm. **d**, Violin plot showing the TIFs of the control and TRF2 knock-down cells. Each dot represents TIFs in a cell. The dots and bars represent means and confidence intervals of the means, respectively, ≥280 cells per condition over three independent experiments. *P* value from a two-tailed unpaired t-test: *** $P < 0.0001$. Source data are provided as a Source Data file.

Supplementary Fig. 6

a

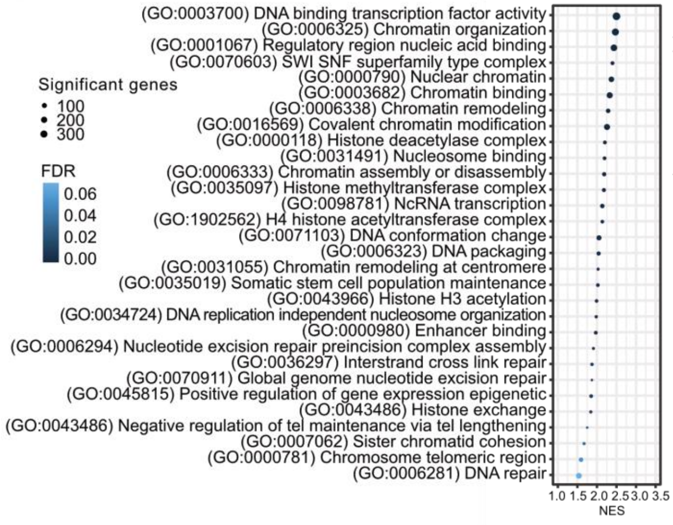

b

GO Term	Count	%	P-value	FDR
Transciprtion, DNA-templated (GO:0006351)	30	30.90%	1.90E-09	9.30E-07
DNA binding (GO:0003677)	26	26.80%	6.60E-07	5.90E-05
Transcription factor activity, sequence-specific DNA binding (GO:0003700)	14	14.40%	2.30E-04	8.00E-03
RNA binding (GO:0003723)	16	16.50%	3.00E-06	1.30E-04
mRNA processing (GO:0006397)	9	9.30%	1.10E-04	1.10E-02
Chromosome, telomeric region (GO:0000784)	8	8.20%	1.80E-06	6.40E-05
Chromosome, centromeric region (GO:0000775)	5	5.20%	3.90E-03	5.00E-02
Chromatin binding (GO:0003682)	6	6.20%	2.70E-04	7.80E-03
Stem cell population maintenance (GO:0019827)	5	5.20%	3.20E-04	2.60E 02

c

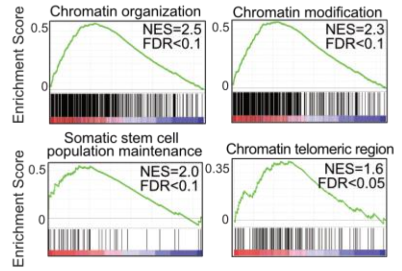

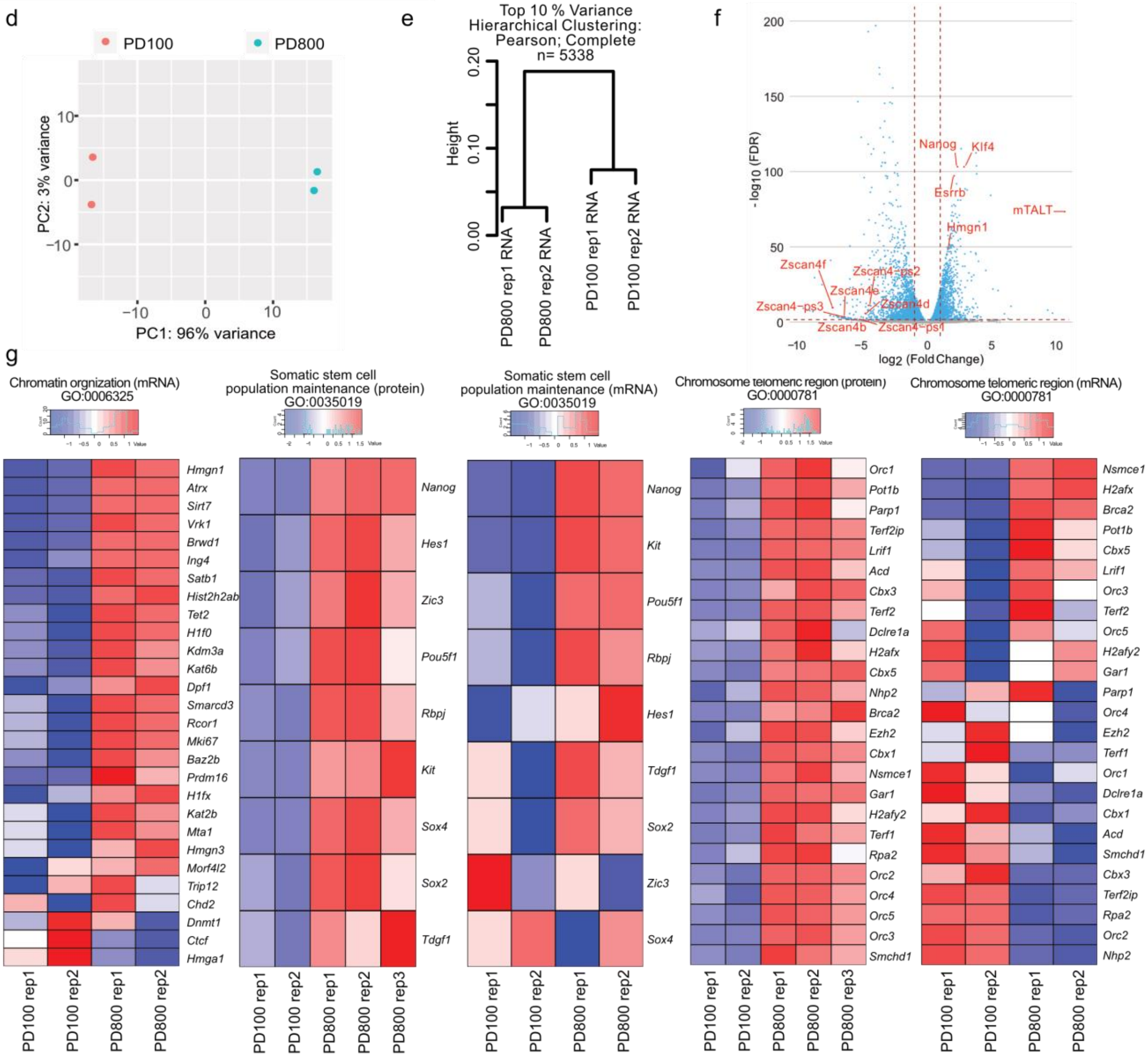

Supplementary Fig. 6

Specific Gene Ontology terms are enriched in ALT mESCs. a, Gene Ontology (GO) analysis of proteomics data with a gene set enrichment assay (GSEA). **b**, GO analysis of proteomics data with a gene set enrichment analysis (DAVID). Enrichment P values were calculated by Fisher's Exact test (modified as EASE score) and adjusted for the FDR (false discovery rate) using the Benjamini–Hochberg adjustment. **c**, Representative enrichment plots of the GSEA of proteomics. **d**, Principal component assay of RNA-seq data of PD100 and PD800 cells. **e**, Hierarchical clustering of RNA-seq peaks. **f**, Volcano plot showing differentially expressed genes in post-ALT cells (PD800) compared with pre-ALT cells (PD100). RNA-seq was done with duplicates of each sample. Vertical lines indicate 1.5 fold change lines, and the horizontal line indicates FDR = 0.1. **g**, Heat map showing the differentially expressed RNA or proteins of the specified terms. Colour index indicates z-score of each expression and ‘count’ (with regard to histogram) indicates the accumulated number of indicated values represented in the heatmap. Source data are provided as a Source Data file.

Supplementary Fig. 7

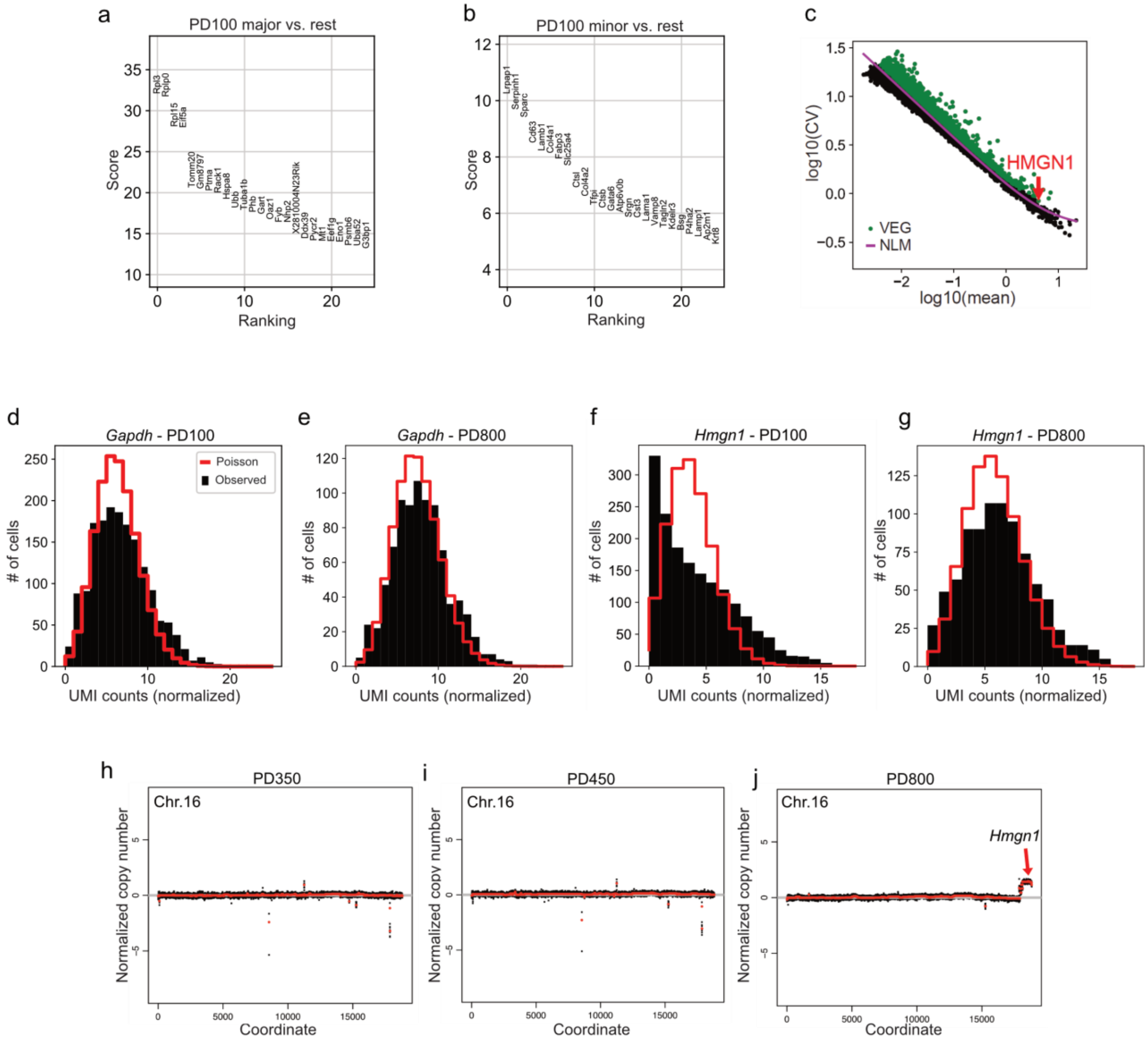

Supplementary Fig. 7

HMGN1 is required for telomere maintenance of alternative lengthening of telomere (ALT) mESCs. a, b, Genes were ranked according to their contributions to differentiating the indicated clusters from the others. **c**, Mean-coefficient variation plot. Individual genes and highly variable genes (FDR <0.05) are labelled by black and green dots, respectively. The purple line represents gene expression variation models based on the negative binomial model. **d-g**, Histogram of UMI counts (black bars) and fitted Poisson counts (red lines) for *Gapdh* and *Hmgn1* at PD100 and PD800. **h-j**, Copy-number variations of chromosome 16 at each passage.

Supplementary Fig. 8

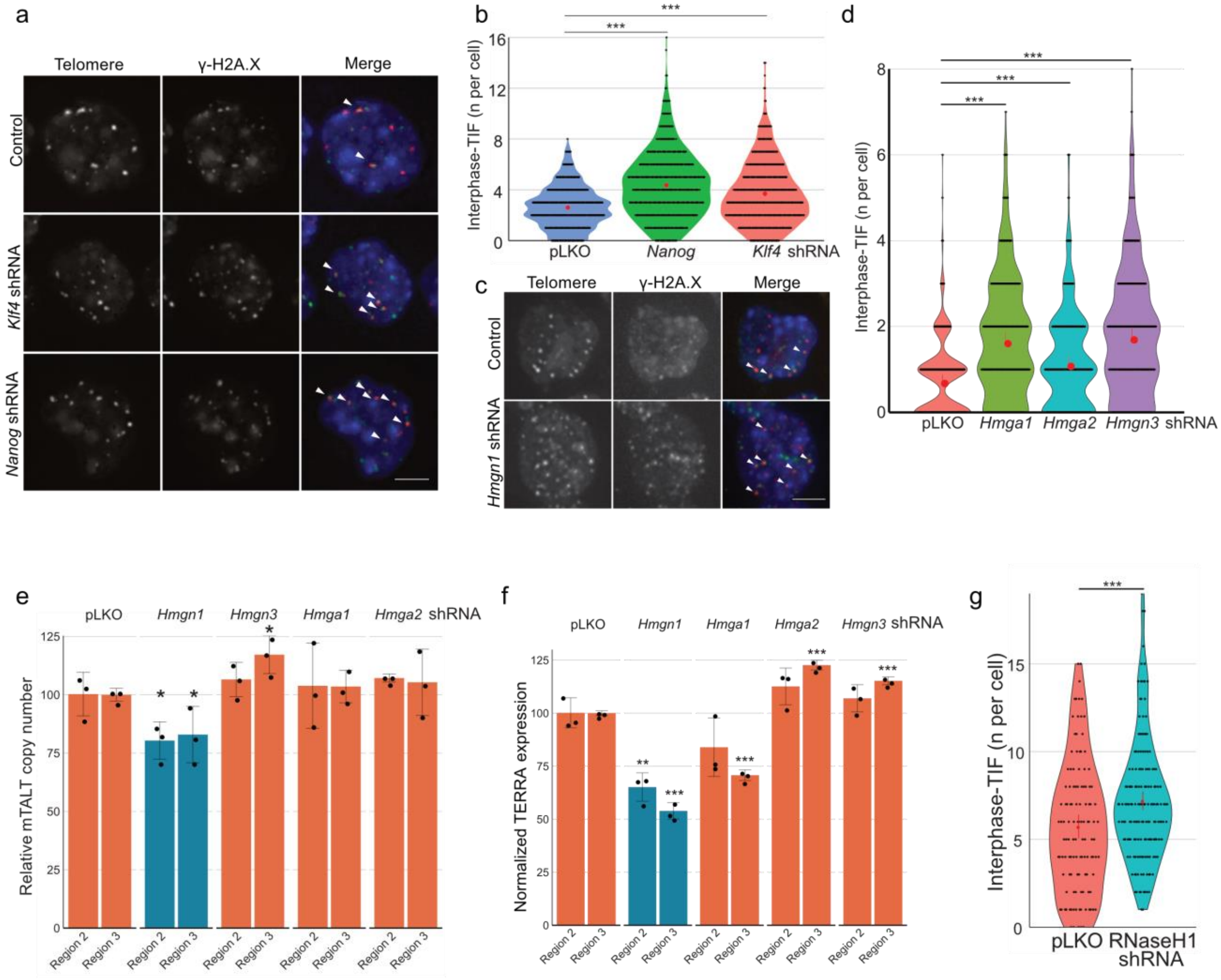

Supplementary Fig. 8

Telomere maintenance is distinctively affected by stemness genes, HMG family genes and RNaseH1. a, Representative images of TIF analysis. Scale bar, 5µm. **b**, Violin plot showing the TIFs of the control, *Klf4* and *Nanog* knock-down cells. Each dot represents TIFs in a cell. The dots and bars represent means and confidence intervals of the means, respectively, from ≥170 cells per condition over three independent experiments. *P* value from a two-tailed unpaired t-test: *** $P < 0.0001$. **c**, Representative image of TIF analysis of control and HMGN1 knock-down cells. Scale bar, 5µm. **d**, Violin plot showing the TIFs of indicated cells. The dots and bars represent means and confidence intervals of the means, respectively, from ≥117 cells per condition over three independent experiments. *P* value from a two-tailed unpaired t-test: *** $P < 0.0001$. **e**, Telomere length quantification with mmqPCR. The bars represent the means and SDs from three biologically independent replicates. P values from a two-tailed unpaired t-test: *Hmgn1* Region 2 * $P = 0.0139$, Region 3 * $P = 0.0256$; *Hmgn3* *$P = 0.0315$. **f**, TERRA expression quantified by qPCR. The bars represent the means and SDs from three biologically independent replicates. *P* values from a two-tailed unpaired t-test: *Hmgn1* Region 2 ** $P = 0.0034$, *** $P < 0.0001$; *Hmga1* Region 3 ***$P < 0.0001$, *Hmga2* *** $P < 0.0001$; *Hmgn3* *** $P = 0.0003$. **g**, Violin plot showing the TIFs of the control and RNaseH1 knock-down cells. Each dot represents TIFs in a cell. The dots and bars represent means and confidence intervals of the means, respectively, from ≥38 cells per condition over three independent experiments. *P* value from a two-tailed unpaired t-test: *** $P < 0.0001$. Region 2 and 3 denote specific regions within the mTALT sequence.
