# Supplementary material for: Telomeres reforged with non-telomeric sequences in mouse embryonic stem cells
Source: Nat Commun. 2021 Feb 17;12:1097. doi: 10.1038/s41467-021-21341-x (PMC7889907; doi:10.1038/s41467-021-21341-x)
Supplement: Supplementary file 6 — Reporting Summary [file 41467_2021_21341_MOESM6_ESM.pdf]

nature research

Corresponding author(s): Junho Lee

Last updated by author(s): Jan 4, 2021

Reporting Summary

Nature Research wishes to improve the reproducibility of the work that we publish. This form provides structure for consistency and transparency in reporting. For further information on Nature Research policies, see our Editorial Policies and the Editorial Policy Checklist.

Statistics

For all statistical analyses, confirm that the following items are present in the figure legend, table legend, main text, or Methods section.

n/a	Confirmed	
☐	☒	The exact sample size (*n*) for each experimental group/condition, given as a discrete number and unit of measurement
☐	☒	A statement on whether measurements were taken from distinct samples or whether the same sample was measured repeatedly
☐	☒	The statistical test(s) used AND whether they are one- or two-sided<br>*Only common tests should be described solely by name; describe more complex techniques in the Methods section.*
☐	☒	A description of all covariates tested
☐	☒	A description of any assumptions or corrections, such as tests of normality and adjustment for multiple comparisons
☐	☒	A full description of the statistical parameters including central tendency (e.g. means) or other basic estimates (e.g. regression coefficient) AND variation (e.g. standard deviation) or associated estimates of uncertainty (e.g. confidence intervals)
☐	☒	For null hypothesis testing, the test statistic (e.g. *F*, *t*, *r*) with confidence intervals, effect sizes, degrees of freedom and *P* value noted<br>*Give P values as exact values whenever suitable.*
☒	☐	For Bayesian analysis, information on the choice of priors and Markov chain Monte Carlo settings
☒	☐	For hierarchical and complex designs, identification of the appropriate level for tests and full reporting of outcomes
☒	☐	Estimates of effect sizes (e.g. Cohen's *d*, Pearson's *r*), indicating how they were calculated

Our web collection on statistics for biologists contains articles on many of the points above.

Software and code

Policy information about availability of computer code

Data collection	softWoRx 6.11 (TIF analysis, CO-FISH), Image Quant LAS 4000 mini (c-circle assay), v.1.2, NovaSeq 6000 (Illumina) (whole genome sequencing, ATAC-seq), HiSeq 4000 (Illumina) (RNA-seq),
Data analysis	R v.3.6.3 (statistics), R ggplot2 v.3.3.0 (graph visualization), R bbplot v.0.2 (graph visualization), UCSC Genome Browser (genomic track), MatCol v.0.95 (colocalisation analysis), Burrows-Wheeler Aligner (BWA) MEM algorithm v.0.7.17 (WGS read mapping), ngCGH v.1 and DNAcopy v.1.52 (copy number calculation), BioCircos v.0.3.4 (circos plot), Haplotyper Caller (SNP and INDEL analysis), DAVID v.6.8 (functional enrichment of genes), MACS2 v.2.1.2 (ATAC-seq peak calling), R/Bioconductor DESeq2 v.1.18.1 (ATAC-seq read count), chromHMM (chromatin state), deepTools v.3.3.0 (average profiles of ATAC-seq peaks), STAR aligner v.2.5.3a (RNA-seq align), RSEM v.1.3.0 (Fragments Per Kilobase Million and Transcripts Per Kilobase Million calculation), DESeq2 v.1.18.1 (differentially expressed genes), SMRT Link v.6.0 (long-read Iso-seq), inDrops pipeline (https://github.com/indrops/indrops, single-cell RNA-seq), Computel v.1.2 (telomere counting), TelomereHunter v.1.1.0 (variant telomere), Telomerecat v.3.2 (pair/single telomere read counting), DAVID v.6.8 (Gene-annotation enrichment analysis), GSEA v.2.0 (gene set enrichment analysis), MSigDB v.7 (proteomics ranking), Scanpy package v. 1.6.0 (single-cell RNA sequencing), BEDTools v.2.25.0 (ATAC-seq anaylsis), ImageJ v.1.53 (Cell growth assay)

For manuscripts utilizing custom algorithms or software that are central to the research but not yet described in published literature, software must be made available to editors and reviewers. We strongly encourage code deposition in a community repository (e.g. GitHub). See the Nature Research guidelines for submitting code & software for further information.

Data

Policy information about availability of data

All manuscripts must include a data availability statement. This statement should provide the following information, where applicable:

- Accession codes, unique identifiers, or web links for publicly available datasets
- A list of figures that have associated raw data
- A description of any restrictions on data availability

The authors declare all data included in this study are available within the paper and the Supplementary files. The ATAC-seq, RNA-seq, whole genome sequencing, and Iso-Seq data are deposited at NCBI GEO: GSE147916 (https://www.ncbi.nlm.nih.gov/geo/query/acc.cgi?acc=GSE147916). Single-cell RNA-seq data are deposited at NCBI GEO: GSE160487 (https://www.ncbi.nlm.nih.gov/geo/query/acc.cgi?acc=GSE160487). The mass spectrometry raw data and searching files have been deposited to the ProteomeXchange Consortium (http://proteomecentral.proteomexchange.org) with the dataset identifier <PXD020419> via MassIVE database (FTP download link: ftp://massive.ucsd.edu/MSV000085772/).
Source data are provided with this paper.

Field-specific reporting

Please select the one below that is the best fit for your research. If you are not sure, read the appropriate sections before making your selection.

☒ Life sciences ☐ Behavioural & social sciences ☐ Ecological, evolutionary & environmental sciences

For a reference copy of the document with all sections, see nature.com/documents/nr-reporting-summary-flat.pdf

Life sciences study design

All studies must disclose on these points even when the disclosure is negative.

Sample size	No statistical methods were used to calculate the sample size. Sample size was determined based on prior studies and literature in the field using similar experiments. Examples include Cesare, A.J. et al. Nat Struct Mol Biol (2009); Arora, R. et al. Nature Communications (2014); Beishline, K. et al. Nature communications (2017).
Data exclusions	No data were excluded.
Replication	Similar results were obtained from at least three biologically independent experiments unless stated otherwise in figure legends. Data from multiple experiments were pooled.
Randomization	No specific randomization method was used. Cells and chromosomes for imaging were selected randomly. Other experiments need not to be randomized because they do not involve group analyses.
Blinding	Investigators were blinded to the generations of mESCs or shRNA treatments when feasible. In addition, investigators were blinded to experimental conditions during image acquisitions and quantifications by a colleague. Other experiments (including various sequencings, mass spectrometry, DRIP, ChIP, and so on) need not to be done under blinding because the correct identification of samples was important for analyses and bias would not affect results.

Reporting for specific materials, systems and methods

We require information from authors about some types of materials, experimental systems and methods used in many studies. Here, indicate whether each material, system or method listed is relevant to your study. If you are not sure if a list item applies to your research, read the appropriate section before selecting a response.

Materials & experimental systems

n/a	Involved in the study	
☐	☒	Antibodies
☐	☒	Eukaryotic cell lines
☒	☐	Palaeontology and archaeology
☒	☐	Animals and other organisms
☒	☐	Human research participants
☒	☐	Clinical data
☒	☐	Dual use research of concern

Methods

n/a	Involved in the study	
☒	☐	ChIP-seq
☒	☐	Flow cytometry
☒	☐	MRI-based neuroimaging

Antibodies

Antibodies used	γ-H2A.X (Biolegend #613401) 1/1000 for immuno-staining, TRF2 (Novus Biologicals #NB110-57130) 1/1000 for immunostaining,

Antibodies used	HMGN1 (produced from Michael Bustin lab, DOI: 10.1038/s41467-018-07687-9) 1/1000 for immunostaining, S9.6 (Kerafast #ENH001) not diluted for DRIP
Validation	γ-H2A.X (Biolegend #613401): Mouse, immunofluorescence. Validated on manufacturer's website: https://www.biolegend.com/en-us/products/purified-anti-h2a-x-phospho-ser139-antibody-1990<br>TRF2 (Novus Biologicals #NB110-57130): Mouse, immunofluorescence. Validated on manufacturer's website: https://www.novusbio.com/products/trf-2-antibody_nb110-57130<br>HMGN1 (a gift from M. Bustin): Mouse, immunofluorescence, ChIP, Validated with personal communications and published references (Bing He et al., Nature Communications, 2018, DOI: 10.1038/s41467-018-07687-9)<br>S9.6 (Kerafast #ENH001): Mouse, DRIP assay. Validated on manufacturer's website: https://www.kerafast.com/productgroup/432/anti-dna-rna-hybrid-s96-antibody

Eukaryotic cell lines

Policy information about cell lines

Cell line source(s)	Terc knock-out mESCs (including all ALT survivor cells) were generated by H. Niida. HEK293FT cell (Takara #632180)
Authentication	We authenticated mESCs using SNP profiling according to Yu et al., Nature (2015) (DOI: 10.1038/nature14397). The paper says ≥ 90% identity score by SNP profiling was recommended for authentication. The identity score using about 2.3 million SNPs for PD100 and PD800 cell lines was 94.28%, indicating that the WGS data ensure the authenticity.
Mycoplasma contamination	Cells were tested negative for mycoplasma.
Commonly misidentified lines (See ICLAC register)	No commonly misidentified cell lines were used.
