# Supplementary material for: Telomeres reforged with non-telomeric sequences in mouse embryonic stem cells
Source: Nat Commun. 2021 Feb 17;12:1097. doi: 10.1038/s41467-021-21341-x (PMC7889907; doi:10.1038/s41467-021-21341-x)
Supplement: Supplementary file 7 — Description of Additional Supplementary Files [file 41467_2021_21341_MOESM7_ESM.docx]

Description of Additional Supplementary Information

Title: Supplementary Data 1.

Description: List of differentially expressed proteins. The list shows differentially expressed proteins in post-ALT, PD800 cells (n=3) compared to pre-ALT, PD100 cells (n=2). Proteins with adjusted P-value lower than 0.1 and absolute fold change higher than 1.5 were considered differentially expressed.

Title: Supplementary Data 2.

Description: List of differentially expressed genes. The list shows differentially expressed genes in post-ALT, PD800 cells (n=2) compared to pre-ALT, PD100 cells (n=2). Genes with adjusted P-value lower than 0.01 and absolute fold change higher than 2 were considered differentially expressed.

Title: Supplementary Data. 3: List of used primers
